# Performance of Matrix-Assisted Laser Desorption Ionization-Time of Flight Mass Spectrometry for Identification of *Scedosporium*, *Acremonium*-Like, *Scopulariopsis*, and *Microascus* Species

**DOI:** 10.3389/fmicb.2022.841286

**Published:** 2022-03-02

**Authors:** Linwei Wei, Jin Shao, Yinggai Song, Zhe Wan, Limin Yao, Hong Wang, Jin Yu

**Affiliations:** ^1^Department of Dermatology and Venereology, Peking University First Hospital, Beijing, China; ^2^Research Center for Medical Mycology, Peking University, Beijing, China; ^3^National Clinical Research Center for Skin and Immune Diseases, Beijing, China; ^4^Beijing Key Laboratory of Molecular Diagnosis on Dermatoses, Beijing, China

**Keywords:** MALDI-TOS MS, identification, *Scedosporium*, *Acremonium*, *Scopulariopsis*, *Microascus*

## Abstract

Matrix-assisted laser desorption ionization-time of flight mass spectrometry (MALDI-TOF MS) has emerged as a powerful microorganism identification tool. Research on MALDI-TOF MS identification of rare filamentous fungi is still lacking. This study aimed to evaluate the performance of MALDI-TOF MS in the identification of *Scedosporium*, *Acremonium*-like, *Scopulariopsis*, and *Microascus* species. Sabouraud broth cultivation and formic acid/acetonitrile protein extraction were used for MALDI-TOF MS identification by a Bruker Biotyper system. An in-house database containing 29 isolates of *Scedosporium*, *Acremonium*-like, *Scopulariopsis*, and *Microascus* spp. was constructed. A total of 52 clinical isolates were identified using the Bruker Filamentous Fungi Library v1.0 (FFL v1.0) alone, and Filamentous Fungi Library v1.0 plus the in-house library, respectively. The mass spectrum profile (MSP) dendrograms of the 28 *Scedosporium* isolates, 26 *Acremonium*-like isolates, and 27 *Scopulariopsis* and *Microascus* isolates were constructed by MALDI Biotyper OC 4.0 software, respectively. The correct species identification rate significantly improved when using the combined databases compared with that when using FFL v1.0 alone (*Scedosporium* spp., 75% versus 0%; *Acremonium*-like spp., 100% versus 0%; *Scopulariopsis* and *Microascus* spp., 100% versus 62.5%). The MSP dendrograms differentiated *Acremonium*-like species, *Scopulariopsis* and *Microascus* species clearly, but cannot distinguish species in the *Scedosporium apiospermum* complex. In conclusion, with an expanded database, MALDI-TOF MS is an effective tool for the identification of *Scedosporium*, *Acremonium*-like, *Scopulariopsis*, and *Microascus* species.

## Introduction

In recent years, with the development of organ transplantation and the widespread use of immunosuppressants and antibiotics, the number of cases of invasive infections caused by filamentous fungi have increased (Brown et al., 2012). In addition to that of infection caused by the most common filamentous fungus, *Aspergillus*, the incidence of infections caused by non-aspergillus filamentous fungi such as *Fusarium*, *Mucorales*, *Scedosporium*, and other rare fungi is also increasing (Miceli and Lee, 2011). *Scedosporium*, *Acremonium*-like, *Scopulariopsis*, and *Microascus* spp. are saprobic fungi commonly found in the environment, some species have been reported as pathogens of humans, and most are opportunistic (Ramirez-Garcia et al., 2018; Perez-Cantero and Guarro, 2020a,b). Within the genus *Scedosporium*, the *Scedosporium apiospermum* species complex and *Scedosporium aurantiacum* are most related to human diseases; the former currently contains *Scedosporium apiospermum*, *Scedosporium boydii*, *Scedosporium ellipsoideum*, *Scedosporium angustum*, and *Scedosporium fusarium* (Ramirez-Garcia et al., 2018). *Aremonium*-like spp. comprise a high diversity of morphologically and genetically related fungi, among which *Aremonium egyptiacum* and *Sarocladium kiliense* are the most commonly involved in human diseases (Perez-Cantero and Guarro, 2020b). *Scopulariopsis*-like spp. include a group of hyaline and dematiaceous fungi, and most of the clinically relevant species belong to the genera *Scopulariopsis* and *Microascus* (Perez-Cantero and Guarro, 2020a). The above pathogenic fungi have raised concern in the field of medical mycology, as they show intrinsic resistance to multiple antifungal drugs (Ramirez-Garcia et al., 2018; Perez-Cantero and Guarro, 2020a,b). Additionally, differences in *in vitro* susceptibility have been reported among species within *Scedosporium* and *Scopulariopsis*-like spp. (Lackner et al., 2012; Yao et al., 2015). Therefore, rapid and accurate identification of these fungi is important for timely treatment, as well as for the supplementation of epidemiological data and drug susceptibility studies of a large range of species. However, due to the taxonomic complexity of these genera and the interspecies morphological similarities, morphological methods are often unable to identify these fungi at the species level (Perdomo et al., 2011; Ramirez-Garcia et al., 2018; Perez-Cantero and Guarro, 2020a). DNA sequencing allows accurate identification but is expensive, labor intensive, time consuming, and not suitable for routine laboratory testing.

Over the last few years, matrix-assisted laser desorption ionization-time of flight mass spectrometry (MALDI-TOF MS) has been increasingly used for the laboratory identification of yeast and filamentous fungi because of its high accuracy, simple operation and low cost (Wilkendorf et al., 2020). The main obstacles to the application of this technique to the identification of filamentous fungi are the lack of a sufficient database and of a rapid and effective protein extraction method (Wilkendorf et al., 2020). Few studies have been conducted on the identification of *Scedosporium* (Del Chierico et al., 2012; Normand et al., 2013; Ranque et al., 2014; Sitterle et al., 2014; Sleiman et al., 2016; Zvezdanova et al., 2019), *Acremonium*-like (Becker et al., 2014; Rychert et al., 2018), *Scopulariopsis* and *Microascus* species (Lau et al., 2013; Becker et al., 2014; Schulthess et al., 2014; Levesque et al., 2015; Riat et al., 2015; Stein et al., 2018; Sacheli et al., 2020) by MALDI-TOF MS. In this study, we developed an in-house database containing 29 strains covering 21 species of *Scedosporium*, *Acremonium*-like, *Scopulariopsis*, and *Microascus* and challenged the system with 52 clinical isolates to evaluate the identification performance of the Bruker Filamentous Fungi Library v1.0 (FFL v1.0) (Bruker Daltonics, Bremen, Germany) alone and FFL v1.0 plus the in-house database.

## Materials and Methods

### Fungal Strains

A total of 81 clinical isolates preserved at the Research Center for Medical Mycology of Peking University were included in this study. The sources and antifungal susceptibility data of these isolates were shown in the previous studies (Wang et al., 2015; Jagielski et al., 2016; Yao et al., 2019). Twenty out of 28 *Scedosporium* isolates (four species) were identified by DNA sequencing of the partial β-tubulin (*BT2*, exons 2–4), calmodulin (*CAL*, exons 3, 4), second large subunit of RNA polymerase II (*RPB2*), superoxide dismutase (*SOD*), and actin (*ACT*) loci in a previous study (Wang et al., 2015). The remaining eight *Scedosporium* isolates were identified by *BT2* locus sequencing using the same method and the GenBank accession numbers of them were MW528357 to MW5283564. Twenty-six *Acremonium*-like isolates (seven species) were identified by sequencing the internal transcribed spacer (ITS), ribosomal large subunit (LSU), and transcription elongation factor 1-α (*EF1-*α) loci in a previous study (Yao et al., 2019). Twenty-seven *Scopulariopsis* and *Microascus* isolates (10 species) were identified by sequencing the ITS, LSU, *TUB*, and *EF1-*α loci as previously reported (Jagielski et al., 2016). Twenty-seven of the 81 isolates were selected as reference strains to build the novel Beijing Medical University (BMU) database (Table 1). Challenge isolates comprising 20 *Scedosporium*, 16 *Acremonium*-like, and 16 *Scopulariopsis* and *Microascus* isolates (Table 1) were used to assess the performance of FFL v1.0 alone versus that of the combined databases for species identification.

**TABLE 1:** Clinical isolates and species used in this study for Beijing Medical University (BMU) database construction and matrix-assisted laser desorption ionization-time of flight mass spectrometry (MALDI-TOF MS) identification assay.

Species	No. of strains used for the BMU database construction	No. of strains used for MALDI-TOF MS identification assay	Total
*Scedosporium* spp.	8	20	28
*Scedosporium boydii*	3	10	13
*Scedosporium apiospermum*	2	6	8
*Scedosporium ellipsoidea*	2	3	5
*Scedosporium angusta*	1	1	2
*Acremonium*-like spp.	10	16	26
*Acremonium egyptiacum*	4	11	15
*Acremonium acutatum*	1	0	1
*Sarocladium terricola*	1	0	1
*Sarocladium strictum*	1	0	1
*Sarocladium kiliense*	1	3	4
*Simplicillium lanosoniveum*	1	1	2
*Lecanicillium* sp. I	1	1	2
*Scopulariopsis* spp.	11	16	27
*Scopulariopsis brevicaulis*	2	11	13
*Scopulariopsis candida*	1	0	1
*Microascus chinensis*	1	1	2
*Microascus gracilis*	1	1	2
*Microascus onychoides*	1	2	3
*Microascus croci*	1	0	1
*Microascus brunneosporus*	1	1	2
*Microascus cirrosus*	1	0	1
*Microascus intricatus*	1	0	1
*Microascus restrictus*	1	0	1
Total	29	52	81

### Sample Preparation

All strains were cultivated on potato dextrose agar (PDA) slants at 28°C for 5–10 days until the colonies reached a size of approximately 1 cm in diameter. The spores and hyphae were scraped by a long, sterile, wet cotton swab; reinoculated in 5 mL of Sabouraud dextrose broth (SDB) (Becton Dickinson, Franklin Lakes, NJ, United States) at 28°C on a Loopster digital rotator (IKA, Staufen, Germany); and incubated for 24–48 h to yield a high quantity of small mycelial balls.

The ethanol/formic acid extraction procedure was performed according to the manufacturer’s instructions. Briefly, 1.5 mL of mycelium was transferred into a 1.5-mL tube (Eppendorf, Hamburg, Germany) after settling and centrifuged at 15,870 × *g* for 2 min. Then, the pellet was washed twice with 1 mL of deionized water. The supernatant was discarded, and then, 300 μL of deionized water and 900 μL of anhydrous ethanol (Sigma-Aldrich, St. Louis, MO, United States) were added and vortexed in sequence. After 10 min of incubation, the materials were centrifuged at 15,870 × *g* for 2 min, and the supernatant was discarded. After drying at 37°C, the pellet was thoroughly mixed in 25–100 μL of 70% formic acid (the volume of 70% formic acid depended on the size of the pellet), incubated for 10 min at ambient temperature, mixed with an equal volume of acetonitrile (Sigma-Aldrich, St. Louis, MO, United States) and incubated for another 10 min. This mixture was centrifuged at 15,870 × *g* for 2 min, and then 1 μL of the supernatant was transferred onto an MTP 384 polished steel MALDI target plate (Bruker Daltonik GmbH, Bremen, Germany), air dried and overlaid with 1 μL of saturated α-cyano-4 hydroxy-cinnamic acid (HCCA) matrix solution (Bruker Daltonics, Bremen, Germany). Finally, the MALDI target was placed into an autoflex speed TOF instrument (Bruker Daltonik, Bremen, Germany).

### Beijing Medical University Database Construction

The reference spectra on *Scedosporium*, *Acremonium*-like, *Scopulariopsis*, and *Microascus* species in FFL v1.0 only include 4 *S. apiospermum* [anamorph] *P. boydii* [teleomorph], 1 *A. strictum* (now named *S. strictum*), 7 S. brevicaulis, 1 *S. acremonium*, and 1 *S. brumptii*. The isolates used for in-house database construction were deposited in eight target spots. The acquisition settings were as follows: ion source 1 at 19.50 kV, ion source 2 at 18.22 kV, lens at 7.01 kV, and mass range from 2,000 to 20,000 Da. The other parameters were kept the default settings. The process of in-house database building was in accordance with Bruker’s procedures. Three fingerprints from each target spot were manually obtained by Bruker Daltonics FlexControl 3.4 software (summing to a signal strength > 10,000); MALDI Biotyper OC 4.0 software was used to evaluate the quality of the 24 fingerprints, poorly quality and poor repeated mass spectra were removed in GelView, and then, 20–24 mass spectra were used to generate a reference main spectrum profile (MSP).

### Matrix-Assisted Laser Desorption Ionization-Time of Flight Mass Spectrometry Identification

The challenge isolates were deposited in four target spots. Spectrum acquisition was performed automatically using MALDI Biotyper RTC 4.0 software (Bruker Daltonik, Bremen, Germany). MALDI Biotyper 3.1 (Bruker Daltonics, Bremen, Germany) was applied for spectral acquisition and comparison with reference spectra from FFL v1.0 and FFL v1.0 plus the BMU database. Identification scores ≥ 2.0 and 1.7–1.99 indicated species- and genus-level identification, respectively, and scores of <1.7 indicated no reliable identification. If the best matches in at least three of the four spots tested were consistent, the MALDI-TOF MS identification was considered interpretable. Correct identification indicates that the results were consistent with the DNA sequencing.

### Composite Correlation Index Matrix

We used the representative composite correlation index (CCI) tool by MALDI Biotyper 4.0 software to analyze the relationships between spectra. CCI value around 1 represents a high conformance of spectra, while that near 0 indicates a clear diversity of the spectra. The CCI matrix translated into a heat map in which closely related spectra are represented in hot colors and unrelated spectra in cold colors.

### Phylogenetic Analysis and Mass Spectrum Profile Dendrograms

Phylogenetic tree of 28 *Scedosporium* isolates was constructed by the *BT2* sequence using the Kimura two-parameter model and the neighbor-joining (NJ) method. Multilocus phylogenetic tree of 26 *Acremonium*-like isolates was constructed by combining the ITS-LSU and *EF1-*α sequences using the Tamura-Nei model and the NJ method. Multilocus phylogenetic tree of 27 *Scopulariopsis* and *Microascus* isolates was constructed by combining the ITS, LSU, *TUB*, and *EF1-*α sequences using the Tamura-Nei model and the NJ method. The bootstrap method (1,000 replicates) was applied to test for phylogeny. The MSP dendrograms of 28 *Scedosporium* isolates, 26 *Acremonium*-like isolates, and 27 *Scopulariopsis* and *Microascus* isolates were constructed by MALDI Biotyper 4.0 software.

### Statistical Analysis

Statistical analysis was performed by SPSS 26. McNemar’s test was used to compare the species identification rates using FFL v1.0 alone and the combined databases, and a *p*-value < 0.05 was considered statistically significant.

## Results

**FIGURE 1:**
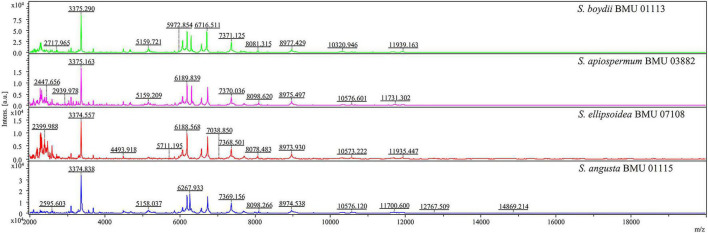
Matrix-assisted laser desorption ionization-time-of-flight mass spectra (m/z 2,000 to 20,000) of *Scedosporium* species.

**FIGURE 2:**
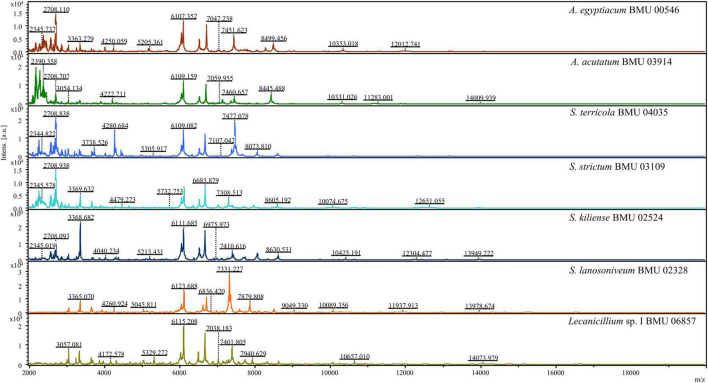
Matrix-assisted laser desorption ionization-time-of-flight mass spectra (m/z 2,000 to 20,000) of *Acremonium*-like species.

**FIGURE 3:**
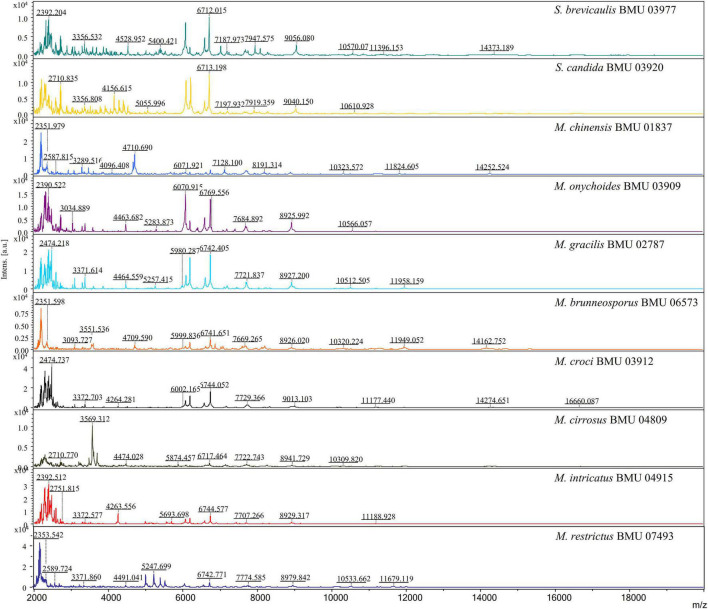
Matrix-assisted laser desorption ionization-time-of-flight mass spectra (m/z 2,000 to 20,000) of *Scopulariopsis* and *Microascus* species.

The mass spectra of *Scedosporium*, *Acremonium*-like, *Scopulariopsis*, and *Microascus* species are shown in Figures 1–3, respectively. The results of MALDI-TOF MS identification of the 52 *Scedosporium*, *Acremonium*-like, *Scopulariopsis*, and *Microascus* isolates by FFL v1.0 alone and by the combined databases are shown in Table 2.

**TABLE 2:** Identification of 52 clinical isolates by the Bruker library and the Bruker library plus BMU database.

Organism (no. of isolates)	No. (%) of isolates identified at the genus or species level by log (score) value							
	
	FFL v1.0				FFL v1.0 plus BMU library			
		
	≥2.0	1.7–1.99	<1.7	Mis-ID	≥2.0	1.7–1.99	<1.7	Mis-ID
*Scedosporium* spp. (20)	0 (0)	7 (35)	1 (5)	12 (60)	15 (75)	0 (0)	0 (0)	5 (25)
*Scedosporium boydii* (10)	0	4	1	5^a^	10	0	0	0
*Scedosporium apiospermum* (6)	0	0	0	6^a^	3	0	0	3^b^
*Scedosporium ellipsoidea* (3)	0	3	0	0	1	0	0	2^c^
*Scedosporium angusta* (1)	0	0	0	1^a^	1	0	0	0
*Acremonium*-like spp. (16)	0 (0)	0 (0)	16 (100)	0 (0)	16 (100)	0 (0)	0 (0)	0 (0)
*Acremonium egyptiacum* (11)	0	0	11	0	11	0	0	0
*Sarucladium kiliense* (3)	0	0	3	0	3	0	0	0
*Simplicillium lanosoniveum* (1)	0	0	1	0	1	0	0	0
*Lecanicillium* sp. (1)	0	0	1	0	1	0	0	0
*Scopulariopsis*-like spp. (16)	10 (63)	1 (6)	5 (31)	0 (0)	16 (100)	0 (0)	0 (0)	0 (0)
*Scopulariopsis brevicaulis* (11)	10	1	0	0	11	0	0	0
*Microascus chinensis* (1)	0	0	1	0	1	0	0	0
*Microascus onychoides* (2)	0	0	2	0	2	0	0	0
*Microascus gracilis* (1)	0	0	1	0	1	0	0	0
*Microascus brunneosporus* (1)	0	0	1	0	1	0	0	0

*Mis-ID, misidentification; FFL, Bruker filamentous fungi library.*

*^a^Identified as Scedosporium apiospermum [anamorph] Pseudallescheria boydii [teleomorph].*

*^b^One misidendified as Scedosporium ellipsoidea, two misidendified as Scedosporium boydii.*

*^c^Misidendified as Scedosporium boydii.*

Using FFL v1.0 alone, 60% (12/20) of *Scedosporium* isolates were identified with scores ≥ 2.0, while all of them were identified as *Scedosporium apiospermum* [anamorph] *Pseudallescheria boydii* [teleomorph], which was the only *Scedosporium* species represented in FFL v1.0, so clear species identification was not available. Seven isolates (35%) were identified to the genus level and also identified as *S. apiospermum* [ana] *P. boydii* [teleo]. Using the combination of FFL v1.0 and the BMU database, the correct species-level identification rates increased significantly to 75% (15/20) (*p* < 0.001), and all 10 *S. boydii* isolates were correctly identified. The remaining five isolates were misidentified as species within the *S. apiospermum* species complex (Table 2).

Using FFL v1.0 alone, none of the *Acremonium*-like isolates were reliably identified. When supplemented with the in-house database, all isolates were correctly identified to the species level. Even though FFL v1.0 contains one strain of *Acremonium strictum* (now named *Sarocladium strictum*), there was no consistent identification of the quadruplicate tests when challenged with one isolate of *S. strictum* used for BMU database construction.

When challenged with *Scopulariopsis* and *Microascus* isolates, FFL v1.0, which contains seven *Scopulariopsis brevicaulis*, one *Scopulariopsis acremonium* and one *Scopulariopsis brumptii* MSPs, identified 62.5% (10/16) and 6.3% (1/16) of the isolates at the species and genus levels, respectively. All of the identified isolates were *S. brevicaulis*. With the combined databases, the species-level identification rate increased significantly to 100% (16/16) (*p* = 0.031).

The species- and genus-levels identification rates of *Scedosporium*, *Acremonium*-like, *Scopulariopsis*, and *Microascus* isolates using the BMU database alone were the same as that using the combined databases.

**FIGURE 4:**
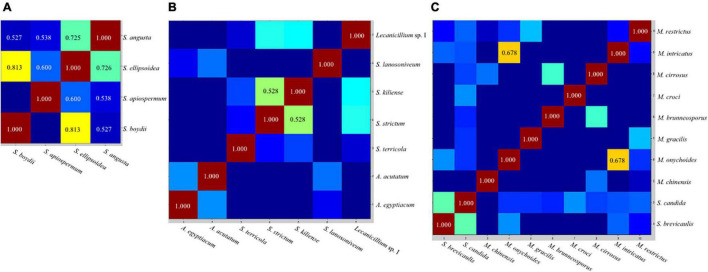
Representative composite correlation index (CCI) matrix derived from *Scedosporium* **(A)**, *Acremonium*-like **(B)**, *Scopulariopsis* and *Microascus* species **(C)**. By comparing the spectra of different species, numerical correlation index is obtained to form the CCI matrix and translated into a heat map. Closely related spectra were represented in hot colors and with higher CCI value, and unrelated spectra in cold colors with lower CCI value. CCI value ≥ 0.5 were shown in the view.

The CCI matrix of test species indicated that mass spectra within the *S. apiospermum* complex were highly similar, while those within *Acremonium*-like, *Scopulariopsis*, and *Microascus* spp. were more diverse (Figure 4).

**FIGURE 5:**
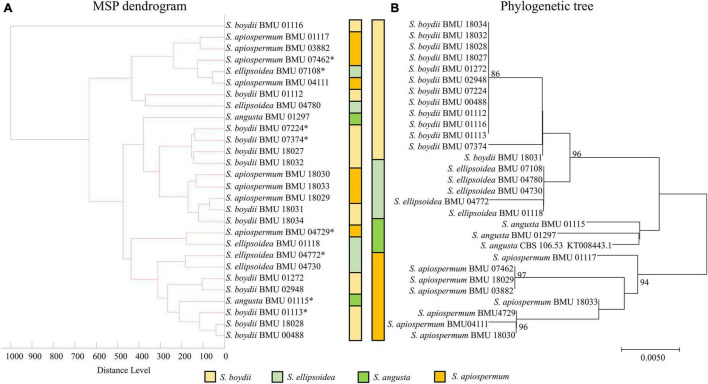
**(A)** Main spectrum profile (MSP) dendrogram of 28 *Scedosporium* isolates based on MALDI-TOF MS analysis; **(B)** molecular phylogenetic tree generated by the Neighbor Joining (NJ) method from the *BT2* sequences of 28 *Scedosporium* isolates.

**FIGURE 6:**
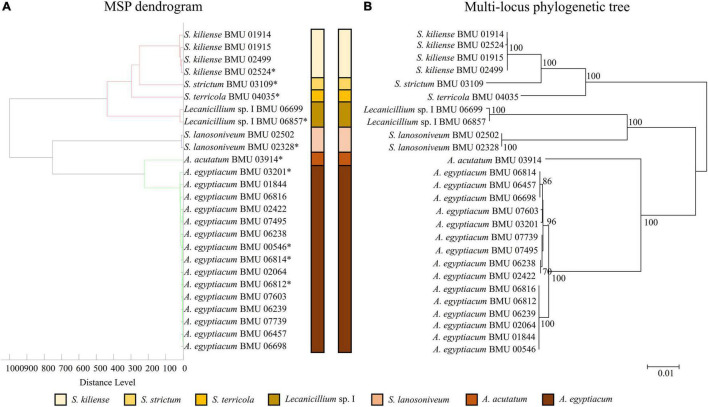
**(A)** Main spectrum profile dendrogram of 26 *Acremonium*-like isolates based on MALDI-TOF MS analysis; **(B)** molecular phylogenetic tree generated by the NJ method from the combined ITS-LSU and *EF1-*α sequences of 26 *Acremonium*-like isolates.

**FIGURE 7:**
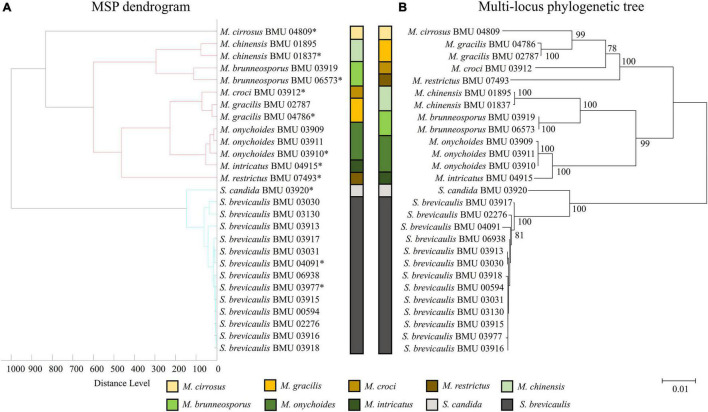
**(A)** Main spectrum profile dendrogram of 27 *Scopulariopsis* and *Microascus* isolates based on MALDI-TOF MS analysis; **(B)** molecular phylogenetic tree generated by the NJ method from the combined ITS, LSU, *TUB*, and *EF1-*α sequences of 27 *Scopulariopsis* and *Microascus* isolates.

The MSP dendrogram of the *Scedosporium* isolates cannot differentiate species in the *S. apiospermum* complex (Figure 5). As for the *Acremonium*-like isolates, the MSP dendrogram showed clear separation of different species, and the topology of the dendrogram appeared to be similar to that of the multilocus phylogenetic tree (Figure 6). The MSP dendrogram of *Scopulariopsis* and *Microascus* spp. clustered 27 isolates to the *Scopulariopsis* clade and *Microascus* clade (Figure 7). Isolates of *M. gracilis* and *M. croci* clustered in one subclade, whereas isolates of *M. onychoides* and *M. intricatus* clustered in another subclade, and the above two pairs of species were also close in the phylogenetic tree. The other isolates belonging to the same species clustered together into respective subclades. However, the topology of the MSP dendrogram and that of the multilocus phylogenetic tree of *Microascus* isolates differed.

## Discussion

The identification of filamentous fungi by MALDI-TOF MS relies mainly on an available database and an efficient protein extraction method. Liquid culture can improve the accuracy of MALDI-TOF MS identification and is usually used for research purpose. In clinical practice, liquid culture can be added when the identification of solid culture is unsatisfactory. The main obstacle of MALDI-TOF MS identification of *Scedosporium*, *Acremonium*-like, and *Scopulariopsis*-like species is a lack of reference spectral data in commercial databases. Our study indicated that MALDI-TOF MS is a powerful technique for rapid and accurate identification of the above species when using the Bruker database complimented with an in-house database and liquid culture.

Due to the non-updated nomenclature and a lack of reference species of *Scedosporium* in FFL v1.0, clear species identification is unavailable. With a supplementary database, the species identification rate of *Scedosporium* spp. ranged between 76 and 100% (Del Chierico et al., 2012; Normand et al., 2013; Ranque et al., 2014; Sleiman et al., 2016; Zvezdanova et al., 2019), and our study also found a correct identification rate of 75% using the combined databases. However, we found that the mass spectra within the *S. apiospermum* complex were highly similar, indicated by the high CCI values between species, so the strains in the complex could not be accurately differentiated, which was also reported by Bernhard et al. (2016). Zvezdanova et al. (2019) also found that even though 9 strains of *S. apiospermum* were included in an in-house database, three strains of *S. apiospermum* were misidentified at the species level (misidentified as *S. boydii* [*n* = 1] or *Lomentospora prolificans* [*n* = 2]). However, Sleiman et al. (2016) found that 17 strains of *Scedosporium* spp. and *L. prolificans* were correctly identified with a supplementary database, but the strains in the *S. apiospermum* complex included only five *S. apiospermum* and one *S. boydii* isolates. Sitterle et al. (2014) found with an in-house database containing 47 reference strains that the Andromas system identified 64 strains of *Scedosporium* spp. (including *S. boydii*, *S. apiospermum*, *S. aurantiacum*, *S. minutispora*, and *S. dehoogii*) and *L. prolificans* to the species level using the direct smearing method (without protein extraction). This indicated that the ability of different MALDI-TOF MS systems to identify *Scedosporium* spp. may be different, and a larger number of strains for database construction may improve the ability of species differentiation. Due to the lack of species, we were unable to clarify whether MALDI-TOF MS can distinguish the *S. apiospermum* complex from other species in the genus. In addition, by comparing the MSP dendrogram with the drug sensitivity data (Wang et al., 2015), we didn’t find a relation between the spectra difference and the drug sensitivity in the test strains. In conclusion, our results showed that MALDI-TOF MS identification for *Scedosporium* spp. by the Bruker Biotyper was relatively reliable after protein extraction from liquid cultivation and database combination.

To date, there have been no MALDI-TOF MS identification studies for a wide range of *Acremonium*-like species. Becker et al. (2014) correctly identified five out of six isolates of *S. strictum* to the species level with a supplemented database by the Bruker Biotyper system. Rychert et al. (2018) correctly identified 30 *Acremonium sclerotigenum* (now named *A. egyptiacum*) (Summerbell et al., 2018) isolates to the species level using the Vitek MS v3.0 database. Due to the limited species in these studies, the identification ability of MALDI-TOF MS for *Acremonium*-like species could not be defined. We conducted the first MALDI-TOF MS identification study on multiple species and genera of *Acremonium*-like spp. Due to a lack of reference MSPs, FFL v1.0 could not identify all of the *Acremonium*-like isolates. Although one *S. strictum* MSP was included in the commercial database, no identification results were found when tested with one *S. strictum* isolate. However, three of the four isolates of *S. kiliense* were identified as *S. strictum*, though with low scores (between 1.464 and 1.671, data not shown), suggesting that the reference strain of *S. strictum* in FFL v1.0 might actually be *S. kiliense*. Some isolates previously reported as *S. strictum* in the literature were also reconfirmed as *S. kiliense* (Perdomo et al., 2011; Summerbell et al., 2018), indicating the possibility of confusion. After supplementation of the BMU database, the identification accuracy by MALDI-TOF MS of *Acremonium*-like species was similar to that of multilocus sequencing, and the former method was faster, simpler and less expensive.

Only a few species and strains of *Scopulariopsis*-like fungi have yet been involved in MALDI-TOF MS identification studies. Using FFL v1.0, *S. brevicaulis* was correctly identified in some cases (Schulthess et al., 2014; Levesque et al., 2015; Riat et al., 2015; Stein et al., 2018; Sacheli et al., 2020), while *S. candida*, *S. cinerea*, *S. brumptii*, and *M. cirrosus* could not be identified (Schulthess et al., 2014; Levesque et al., 2015; Stein et al., 2018). The identification of *Scopulariopsis*-like fungi can be improved by constructing an in-house database (Lau et al., 2013; Becker et al., 2014). Lau et al. (2013) correctly identified three strains of *S. brevicaulis* to the species level using the Bruker Biotyper system plus the self-built NIH database. Becker et al. (2014) also correctly identified 12 strains of *S. brevicaulis* and one strain of *S. candida* with an in-house database. Our study found that FFL v1.0 performed well in the identification of *S. brevicaulis* but was unable to identify other species due to a lack of reference MSPs. After adding the reference MSPs of ten species, the Bruker Biotyper system was able to accurately identify *Scopulariopsis* and *Microascus* species.

The MSP dendrogram obtained by MALDI-TOF MS analysis indicates the relationship between the strains based on protein fingerprint differences and can be applied to the classification of microorganisms. This study found that the MSP dendrogram of *Acremonium*-like isolates clearly distinguished 7 different species, with similar topology to the multilocus phylogenetic tree. The MSP dendrogram of *Scopulariopsis* and *Microascus* spp. clustered isolates of the same species or close species to a branch, while the topology of *Microascus* spp. was inconsistent with that of the multilocus phylogenetic tree. These results indicated that the MSP dendrogram is efficient for species differentiation; however, there was a difference between molecular taxonomy at the nucleic acid and protein levels, which may be due to the varied protein expression related to the growth condition and life cycle of the fungi (Putignani et al., 2011). Shao et al. (2018) also found that the interspecific discrimination of MSP dendrograms and ITS-based trees of Rhizopus and Mucor species was consistent, while there were inconsistences at the intergeneric and intraspecific levels. Research on *Lichtheimia* species by Schrodl et al. (2012) showed that the MSP dendrogram clearly discriminated different species, but the topology was different from that of the phylogenetic tree. Therefore, MSP dendrogram is an excellent tool for interspecific differentiation but is limited in phylogenetic analysis (Shao et al., 2018). In addition, we found the MSP dendrogram cannot differentiate species in the *S. apiospermum* complex. Shao et al. (2020) also found that the MSP dendrogram was difficult to distinguish species in the *Trichophyton mentagrophytes* series. These results indicated discrimination of close species in some species complex by MSP dendrogram is difficult.

This study showed that with the supplementary database, MALDI-TOF MS represents a powerful tool to identify *Scedosporium*, *Acremonium*-like, *Scopulariopsis*, and *Microascus* species. As the test strains were all previously identified storage strains, and the species were rare and difficult to collect in a short time, blind test was not conducted. In the future, the identification effect will be clearer on the basis of continuous database expansion and amplification of test species and strains.

## Data Availability Statement

The datasets presented in this study can be found in online repositories. The names of the repository/repositories and accession number(s) can be found below: https://www.ncbi.nlm.nih.gov/genbank/, MW528357 to MW5283564.

## Author Contributions

JY designed the study. ZW collected the samples. LW, JS, YS, LY, and HW participated in the performance of the research. LW participated in the writing of the manuscript and data analysis. All authors contributed to the article and approved the submitted version.

## Conflict of Interest

The authors declare that the research was conducted in the absence of any commercial or financial relationships that could be construed as a potential conflict of interest.

## Publisher’s Note

All claims expressed in this article are solely those of the authors and do not necessarily represent those of their affiliated organizations, or those of the publisher, the editors and the reviewers. Any product that may be evaluated in this article, or claim that may be made by its manufacturer, is not guaranteed or endorsed by the publisher.
